# Clinical Outcome Data of Children Treated with Cannabis-Based Medicinal Products for Treatment Resistant Epilepsy—Analysis from the UK Medical Cannabis Registry

**DOI:** 10.1055/a-2002-2119

**Published:** 2023-01-30

**Authors:** Simon Erridge, Carl Holvey, Ross Coomber, Jonathan Hoare, Shaheen Khan, Michael W. Platt, James J. Rucker, Mark W. Weatherall, Sushil Beri, Mikael H. Sodergren

**Affiliations:** 1 Department of Surgery & Cancer, Imperial College London, London, United Kingdom; 2 Department of Medicine, Sapphire Medical Clinics, London, United Kingdom; 3 Department of Trauma & Orthopaedics, St Georges NHS Healthcare Trust, London, United Kingdom; 4 Department of Palliative Medicine, Guy's & St Thomas' NHS Foundation Trust, London, United Kingdom; 5 Department of Psychological Medicine, Institute of Psychiatry, Psychology & Neuroscience, Kings College London, London, United Kingdom; 6 Centre for Affective Disorders, South London & Maudsley NHS Foundation Trust, London, United Kingdom; 7 Department of Neurology, Buckinghamshire Healthcare NHS Trust, Amersham, United Kingdom

**Keywords:** pediatrics, epilepsy, treatment-resistant epilepsy, cannabidiol, tetrahydrocannabinol, cannabis

## Abstract

**Background** There is a paucity of high-quality evidence of the efficacy and safety of cannabis-based medicinal products in treatment of treatment-resistant epilepsy (TRE) in children.

**Methods** A case series of children (<18 years old) with TRE from the UK Medical Cannabis Registry was analyzed. Primary outcomes were ≥50% reduction in seizure frequency, changes in the Impact of Pediatric Epilepsy Score (IPES), and incidence of adverse events.

**Results** Thirty-five patients were included in the analysis. Patients were prescribed during their treatment with the following: CBD isolate oils ( *n* = 19), CBD broad-spectrum oils ( *n* = 17), and CBD/Δ ^9^ -THC combination therapy ( *n* = 17). Twenty-three (65.7%) patients achieved a ≥50% reduction in seizure frequency. 94.1% ( *n* = 16) of patients treated with CBD and Δ ^9^ -THC observed a ≥50% reduction in seizure frequency compared to 31.6% ( *n* = 6) and 17.6% ( *n* = 3) of patients treated with CBD isolates and broad-spectrum CBD products, respectively ( *p* < 0.001). Twenty-six (74.3%) adverse events were reported by 16 patients (45.7%). The majority of these were mild ( *n* = 12; 34.2%) and moderate ( *n* = 10; 28.6%).

**Conclusion** The results of this study demonstrate a positive signal of improved seizure frequency in children treated with Cannabis-based medicinal products (CBMPs) for TRE. Moreover, the results suggest that CBMPs are well-tolerated in the short term. The limitations mean causation cannot be determined in this open-label, case series.

## Introduction

Epilepsy, as defined by the International League Against Epilepsy, is a disease where an individual is at a persistently elevated risk for an unprovoked epileptic seizure, having already experienced at least one unprovoked seizure. 1 Epilepsy affects approximately 65 million individuals globally. 2 Specifically, in children and adolescents under 19 years of age, the prevalence is 7.24 cases per 1,000 people. 3 Patients with epilepsy are at an increased risk of morbidity and mortality secondary to direct or indirect effects of seizures. Direct causes include sudden unexpected death in epilepsy (SUDEP), status epilepticus, and trauma, whilst indirect causes include psychological disease, pulmonary aspiration, and the adverse effects of anti-seizure medication. 4 Epilepsy is also associated with reduced quality of life and has a significant social impact on individuals, including educational attendance, stigmatization, discrimination, and social isolation. 5

Whilst strides have been made in the pharmaceutical management of epilepsy, approximately one-third of patients will fail to achieve seizure control despite an adequate trial of optimum anti-seizure medications. 6 For patients with treatment-resistant epilepsy (TRE) the most successful treatment to achieve seizure control is surgical management, yet few patients are suitable candidates for surgery. 7 Consequently, there is a large unmet need to develop novel treatment modalities for TRE. The endocannabinoid system has been implicated in the modulation of glutamate and γ-aminobutyric acid (GABA) neurotransmission via retrograde signalling. 8 Endocannabinoids, such as anandamide and 2-arachidonoylglycerol (2-AG), activate pre-synaptic type 1 cannabinoid receptors (CB1) which reduces calcium influx and subsequent release of GABA and glutamate. 8 This consequently affects both neuronal hyperexcitability and baseline seizure thresholds. 9 Cannabis-based medicinal products (CBMPs) have therefore been identified as treatments in the setting of TRE. In animal models of epilepsy, agonists of CB1 and cannabinoid receptor type 2 (CB2) have demonstrated anti-seizure effects. 10 Endocannabinoids have a complex role in animal models of epilepsy. They have each demonstrated anticonvulsant effects via CB1 and CB2 agonism, whilst 2-AG also directly activates GABA _A_ receptors. 11 They also have pro-convulsant effects due to transient receptor potential cation channel subfamily V member 1 (TRPV1) receptor activation. 11 Cannabidiol (CBD), a phytocannabinoid, has demonstrated clinical efficacy in reducing seizure frequency in certain populations with TRE. 12 Its exact anti-convulsant mechanism is unclear, although it is likely to be multifactorial. Its primary actions are thought to be secondary to reducing intracellular influx of calcium through increasing endogenous anandamide, desensitization of TRPV1 receptors, and blockade of T-type calcium ion channels. 9 13 However, translation into clinical research has been limited. A 2018 systematic review and meta-analysis identified just four randomized controlled trials of 550 patients examining the effects of CBD as an adjunctive treatment in patients with TRE in the setting of Lennox–Gastaut and Dravet syndromes. 14 This found a significant reduction in seizure frequency of 19.9% in those patients on 20 mg/kg/d of CBD, with 37.2% of patients experiencing a reduction in seizure frequency of at least 50%. 14 There is also evidence of a reduction in seizure frequency in patients with tuberous sclerosis treated with CBD as an adjunctive treatment for TRE. 15

Epidyolex (GW Pharmaceuticals, Cambridge, UK), a CBD isolate oil preparation, has now received approval from the European Medicines Agency and the U.S. Food and Drug Administration as an adjunctive treatment for TRE in patients with Lennox–Gastaut and Dravet syndromes. 16 There is a range of clinical trials and observational data describing the effectiveness of CBD isolates in other conditions. 17 Due to their limited scope, these often lack generalizability to clinical practice.

Whilst there are clear anticonvulsant properties of CBD, not all patients respond to therapy with a CBD isolate whilst others achieve some clinical benefit, but still experience a high seizure frequency. 14 15 17 As such, there has been interest in utilizing broad-spectrum CBMPs including delta-9-tetrahydrocannabinol (Δ ^9^ -THC), a partial agonist of CB1 and CB2 receptors, for these patients. 18 Despite biological plausibility, the effect of Δ ^9^ -THC on seizures is still unclear, with some studies suggesting an anticonvulsant effect, whilst others demonstrate pro-convulsant effects. 18 19 20 The interaction of Δ ^9^ -THC and CBD when administered together in pre-clinical models suggests a complex dose–response relationship on individual receptors. 20 Δ ^9^ -THC and other cannabinoids, such as cannabigerol, may also affect the pharmacokinetic properties of CBD leading to a purported entourage effect. 21 To date, there are no randomized controlled trials of Δ ^9^ -THC in the setting of TRE in children. In an observational study, based in Colorado, a broad spectrum of different CBMPs found improvement in seizure frequency in 57% of children and adolescents, with 33% achieving a >50% reduction in seizures. 22 An Israeli case series of children treated with an oil-based CBD and Δ ^9^ -THC preparation, with concentrations of 20 mg/mL and 1 mg/mL, respectively, demonstrated an 89% reduction in overall seizures with 58% of patients having a reduction of ≥50% in seizure frequency. 23

The long-term efficacy and safety of CBMPs for children with TRE are still unclear. The UK Medical Cannabis Registry has been capturing real-world data on patients prescribed CBMPs since 2019 to bridge the gap between clinical practice and the paucity of evidence for medical cannabis. 24 This study aims to assess the response to CBMPs in children with TRE in respect to seizure frequency, validated patient-reported outcome measures (PROMs), and safety.

## Methods

### Study Design

A case series was analyzed in all children with TRE treated with CBMPs in the United Kingdom who were enrolled in the UK Medical Cannabis Registry up until January 2 ^nd^ , 2022. The UK Medical Cannabis Registry is a dedicated registry for the collection of outcomes in patients prescribed CBMPs in the United Kingdom and Channel Islands since 2019. 24 It is wholly managed by Sapphire Medical Clinics. Patients are enrolled on the registry following the provision of informed consent.

### Setting and Participants

The study included patients aged less than 18 years old with TRE who have been prescribed CBMPs. TRE was defined according to the consensus statement of the International League Against Epilepsy as failure of an adequate trial of two or more appropriate and tolerated anti-seizure medications to cause prolonged seizure freedom. 25 Patients were excluded if follow-up was less than 3 months. All CBMPs were initiated by a pediatric neurologist at Sapphire Medical Clinics, supported by a multidisciplinary team of clinicians for other specialties in accordance with UK guidance. 26 These included oil-based isolated cannabinoids or broad/full-spectrum products including cannabinoids, terpenes, and flavonoids. No children were prescribed dry flower preparations. Each medicine was produced in accordance with good manufacturing practice, meaning that they meet regulations on potential contaminants whilst ensuring consistency in the concentration of active cannabinoids. 27 The typical pathway for initiating CBMPs as a treatment for children with TRE is to start with a CBD isolate and assess for treatment response. If the response is inadequate, then a broad-spectrum CBD product is considered. Finally, if the response is still assessed as being insufficient then a trial incorporating a low-dose Δ ^9^ -THC product as an adjunct was considered.

### Data Collection

Demographic data on patients were recorded on initial assessment, including age, sex, and weight. Supplementary co-morbidities were recorded, including learning difficulties and autism. 28 Cannabis consumption that was obtained illicitly was recorded at baseline. As previously described by our group, the lifetime consumption of cannabis was detailed in cannabis gram years. 24

Information on previous and current anti-seizure medications was recorded at baseline. In addition, details on CBMP prescription were recorded including licensed producer, formulation, and highest tolerated dose of CBD and Δ ^9^ -THC per day according to weight (mg/kg/d).

For each patient, the average seizure frequency per month was recorded. This was used to calculate the change in seizure frequency in response to treatment. The percentage of patients with ≥50% and ≥90% reduction in seizure frequency were calculated in line with the benchmarks set in existing clinical trials for response to anti-seizure medications facilitating comparison to existing literature. 29

In addition to clinically derived data collection, patient-reported outcome measures are captured through remote data collection at baseline, 1 month, 3 months, and 6 months. Carers, guardians, and/or parents were asked to complete the Impact of Pediatric Epilepsy Score (IPES). The IPES is a validated clinical and research measure to assess the global effect of epilepsy on both children and their families across health, social, and academic domains. 30 The score consists of 11 questions assessing the impact on specific aspects of living with impact severity graded between 0 (not a lot) and 3 (a lot). The total scale range is therefore from 0 to 33, with a higher score suggestive of worse epilepsy-specific quality of life. 30

Adverse events were also captured during clinical consultations and contemporaneously with the IPES. The severity of adverse events was reported according to the Common Terminology Criteria for Adverse Events v.4.0. 31

### Statistical Analysis

Demographics, comorbidities, previous medications, current doses of CBMPs, and adverse events are displayed as mean (±standard deviation [ ± SD]), median (range), or frequency (%) as appropriate. These were analyzed utilizing descriptive statistics. The Shapiro-Wilk test was utilized to assess the distribution of non-categorical data. Differences in seizure frequency, presented with 95% confidence intervals (95% CI), and adverse events according to CBMP were analyzed utilizing a Chi-square test. The change in IPES between baseline and follow-up periods were analyzed utilizing paired *t* -tests as the data was normally distributed. Statistical significance was determined by *p* -value <0.050. Statistical analysis was performed utilizing Statistical Package for Social Sciences (SPSS) [IBM Statistics version 27 SPSS Inc., (New York, Illinois), United States].

## Results

Thirty-nine patients were identified from the UK Medical Cannabis Registry having been treated with CBMPs for TRE. Thirty-five patients were included in the analysis, with four patients excluded for insufficient (<3 months) follow-up. The demographics of the participants are detailed in Table 1 . The mean age of patients was 9.7 (±4.5) years. Thirty-one (88.6%) participants also had learning difficulties, with nine (25.7%) affected by autism. Three (8.6%) patients were consuming cannabis obtained illicitly before initiation of treatment. The median follow-up duration was 12.0 (3.0–28.0) months.

**Table 1:** Demographic characteristics of participants

Demographics	*N* (%) / mean (±standard deviation) / median (range)
Gender	
Female	15 (42.9%)
Male	20 (57.1%)
Age	9.71 ± 4.50
Weight (kg)	32.35 ± 17.12
Charlson co-morbidity score	0 (0–2)
Learning difficulties	31 (88.6%)
Autism	9 (25.7%)
Cannabis status (Illicitly sourced)	
Current user	3 (8.6%)
Ex-user	0 (0.0%)
Never user	32 (91.4%)

Twelve (34.3%) patients had a diagnosed epilepsy syndrome (Lennox-Gastaut syndrome *n* = 5; Dravet syndrome *n* = 2; all other syndromes *n* = 1 and redacted to maintain anonymity), whilst nine (25.7%) had a genetic cause for their epilepsy. The remaining etiologies ( *n* = 14; 40.0%) were either idiopathic or not known. The most common predominant seizure type experienced by participants were generalized seizures ( *n* = 21; 60.0%), whilst other predominant seizure types also included complex partial seizures ( *n* = 5; 14.3%), simple partial seizures ( *n* = 3; 8.6%), multifocal seizures ( *n* = 3; 8.6%), and unclassified seizures ( *n* = 3; 8.6%). Patients had used a median of 7 (3–15) anti-seizure medications before the initiation of CBMP therapy. They were currently using a median of 2 (1–4) anti-seizure medications concurrently alongside CBMPs. The most commonly co-administered anti-seizure medications were clobazam ( *n* = 17; 48.6%), valproate ( *n* = 11; 31.4%), and lamotrigine ( *n* = 5; 14.3%). The median baseline seizure frequency per month was 120 (2–18,000).

### CBMP Dosing

**Fig. 1:**
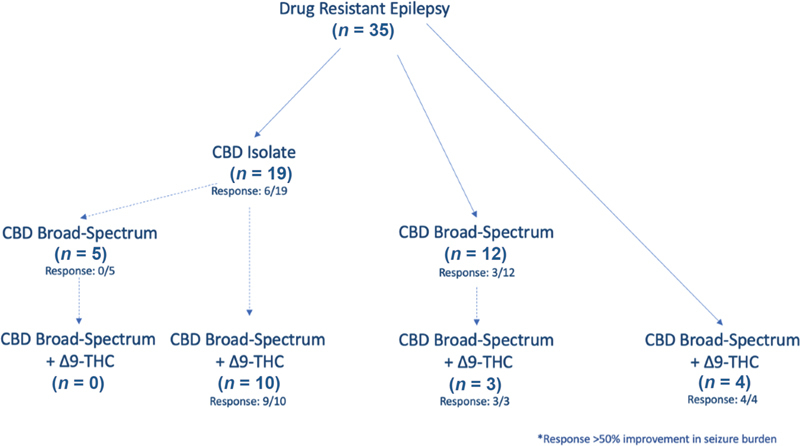
Pathways of treatment with CBMPs from initiation. The response is recorded where a >50% reduction in seizures has occurred. All patients initiated with CBD and Δ ^9^ -THC combination therapy at onset had already initiated treatment outside of Sapphire Medical Clinics, before enrolment into the UK Medical Cannabis Registry. CBD, cannabidiol; CBMP, cannabis-based medicinal products; Δ ^9^ -THC, delta-9-tetrahydrocannabinol.

Patients were prescribed CBMPs from initiation until the date of data extraction in accordance with the pathways as outlined in Fig. 1 . In total, 19 patients had received CBD isolate therapy, whilst 17 patients had been treated with CBD broad-spectrum or CBD and Δ ^9^ -THC combination therapies, respectively. At the time of data extraction, four patients were being treated actively with CBD isolate therapy, whilst 14 and 17 patients were being treated with CBD broad-spectrum or CBD and Δ ^9^ -THC combination therapies, respectively. The maximum tolerated mean daily doses for each therapy are outlined in Supplementary Table S1 (available in online version only). At date of extraction the most prescribed CBD isolate preparation was Adven 150 mg/mL CBD isolate oil (Curaleaf International, Guernsey, UK) ( *n* = 3; 75%). The most administered CBD broad-spectrum preparation was Adven 50 mg/mL CBD broad spectrum oil (Curaleaf International, Guernsey, UK) ( *n* = 21; 67.7%). Bedrolite 10% CBD/< 1% Δ ^9^ -THC (Bedrocan International, Veendam, Netherlands) ( *n* = 6; 35.3%) was the most frequently prescribed CBMP containing Δ ^9^ -THC ( Supplementary Table S2 , available in online version only). The median lengths of treatment with CBD isolate, CBD broad-spectrum, and CBD/Δ ^9^ -THC combination therapy were 8.5 (1–16), 4 (3–8), and 12 (3–27) months, respectively.

### Change in Seizure Frequency

Twenty-three (65.7%; 95% CI: 47.8–80.9%) patients achieved a ≥50% reduction in seizure frequency, whilst 13 (37.1%; 95% CI: 21.5–55.1%) sustained a ≥90% reduction in seizure frequency across all treatment cohorts. Four (11.4%; 95% CI: 3.2–26.7%) patients had complete remission. Sixteen (94.1%; 95% CI: 71.3–99.9%] patients treated with CBD and Δ ^9^ -THC observed a ≥50% reduction in seizure frequency. This was statistically significant compared to patients treated with CBD isolates ( *n* = 6; 31.6%; 95% CI: 12.6–56.6%) and broad-spectrum CBD products ( *n* = 3; 17.6%; 95% CI: 3.8–43.4%), respectively ( *p* <0.001) ( Table 2 ).

**Table 2:** Change in seizure frequency with each treatment modality

Response to treatment with CBMPs	Maximum titrated therapy ( *n* = 35)	CBD isolate ( *n* = 19)	CBD broad spectrum ( *n* = 17)	CBD & Δ ^9^ -THC ( *n* = 17)	*p* -Value
≥50% reduction in seizure frequency	23 (65.7%) [95% CI: 47.8–80.9%]	6 (31.6%) [95% CI: 12.6–56.6%]	3 (17.6%) [95% CI: 3.8–43.4%]	16 (94.1%) [95% CI: 71.3–99.9%]	<0.001
≥90% reduction in seizure frequency	13 (37.1%) [95% CI: 21.5–55.1%]	3 (15.8%) [95% CI: 3.4–39.6%]	3 (17.6%) [95% CI: 3.8–43.4%]	9 (52.9%) [95% CI: 27.8–77.0%]	0.023
Remission	4 (11.4%) [95% CI: 3.2–26.7%]	1 (5.3%) [95% CI: 0.1–26.0%]	0 (0.0%) [95% CI: 0.0–19.5%]	3 (17.6%) [95% CI: 3.8–43.4%]	0.134

Abbreviations: 95% CI, 95% confidence interval; CBD, cannabidiol; CBMPs, cannabis-based medicinal products; Δ ^9^ -THC, delta-9-tetrahydrocannabinol

### Impact of Pediatric Epilepsy Score

Follow-up data with an IPES score was available for 22 patients ( Table 3 ). There was no significant difference in IPES score between baseline and any follow-up period ( *p* >0.050). There was no significant difference in the mean difference of IPES scores according to those who had already commenced therapy before enrolment in the UK Medical Cannabis Registry ( *p* >0.050) ( Supplementary Table S3 , available in online version only). There was also no significant difference in the change in IPES between those treated with CBD isolate, CBD whole spectrum, or CBD and Δ ^9^ -THC combination therapy at any time point ( *p* >0.050) ( Supplementary Table S4 , available in online version only).

**Table 3:** Paired comparison of impact of pediatric epilepsy score between baseline and follow-up assessment

Follow-up month	*n*	Baseline IPES score	Follow-up IPES Score	*p* -Value
Month 1	22	26.8 ± 5.7	24.4 ± 9.3	0.103
Month 3	12	25.2 ± 6.5	24.1 ± 9.7	0.660
Month 6	9	23.9 ± 6.5	20.2 ± 9.7	0.119

Abbreviation: IPES, Impact of Pediatric Epilepsy Score.

### Adverse Events

Table 4 displays the incidence of adverse events according to symptom and severity. All adverse events were reported by 16 patients (45.7%). There was no significant difference in the proportion of patients who experienced adverse effects between those currently prescribed CBD isolates ( *n* = 2; 50%), CBD broad-spectrum products ( *n* = 6; 42.9%), and both CBD and Δ9-THC products ( *n* = 8; 47.1%; *p* = 0.957). A total of 26 (74.5%) adverse events were experienced across the participants. The majority of these were mild ( *n* = 12; 34.2%) and moderate ( *n* = 10; 28.6%). The full adverse effect profile for participants treated with CBD and Δ9-THC CBMPs is detailed in Supplementary Table S5 (available in online version only). Overall, 11 (64.7%) adverse effects were experienced by this patient group. Similar to the frequency across all patient groups, the majority were mild ( *n* = 5; 29.4%) and moderate ( *n* = 4; 23.5%).

**Table 4:** Adverse events reported by participants ( *n* = 39)

Adverse events	Mild	Moderate	Severe	Debilitating/Life threatening	Total
Anorexia	0	0	1	0	1 (2.9%)
Anxiety	0	1	0	0	1 (2.9%)
Constipation	1	0	0	0	1 (2.9%)
Fatigue	11	3	0	0	14 (40.0%)
Lethargy	0	1	0	0	1 (2.9%)
Nausea	0	0	1	0	1 (2.9%)
Seizure frequency increased	0	2	0	0	2 (5.7%)
Sepsis	0	0	0	1	1 (2.9%)
Somnolence	0	2	0	0	2 (5.7%)
Vomiting	0	1	0	0	1 (2.9%)
Weight loss	0	0	1	0	1 (2.9%)
Total	12 (34.2%)	10 (28.6%)	3 (8.6%)	1 (2.9%)	26 (74.3%)

## Discussion

This limited case series represents an analysis of unlicensed CBMPs for children with TRE in Europe, providing initial insights to guide further research and carefully considered clinical practice. Across all patients, 65.7% had a ≥50% reduction in seizure frequency. 94.1% of those prescribed both CBD and Δ9-THC sustained a ≥50% reduction in seizure frequency, significantly higher than treatment without Δ9-THC ( *p* = 0.003). This compares very favorably with the quoted incidence of ≥50% seizure reduction between 14.3 and 54.0% for anti-seizure medications in treatment-resistant focal epilepsy. 32 Adverse events were experienced by 45.7% of patients. 15.4% of adverse events were categorized as severe or worse. There was no significant difference in the incidence of adverse events between any treatment regimen ( *p* = 0.957). These results must be viewed in the context of a strong placebo effect previously described in randomized controlled trials for anti-seizure medications for TRE in adults and children, whereby the pooled incidence of ≥50% reduction of seizures in control groups is 15%. 33

The reduction in seizure frequency observed in this study is similar to that reported previously. The largest series in the United States of 75 patients reported 33% attaining a >50% reduction in seizures. 22 This is comparable to the 65.7% of patients who observed a similar reduction in the present study. A study of 74 children with TRE limited to a CBD-enriched CBMP with a ratio of CBD:Δ ^9^ -THC of 20:1 observed 58% of patients having a reduction of >50% in seizure frequency. 23 The seizure response across each study may be reflective of the proportion of patients treated with therapies containing Δ9-THC, with those studies utilizing Δ9-THC in a higher proportion of patients having an increased incidence of response ≥50%. Treatment with Δ9-THC was found to be associated with a statistically significant seizure reduction compared to just CBD isolate or CBD whole plant extract in the present study ( *p* < 0.001). The placebo effect of CBMPs, particularly Δ9-THC, is noted to be strong considering its psychoactive and vasoactive effects in addition to smell, taste, 14 34 and the expectation of clinical effect. 35 As such trials examining CBMPs for TRE have seen higher rates of reduction in seizure frequency in placebo arms compared to trials examining other anti-seizure medication. 14 34 This may be a causative mechanism in the present study's results.

With respect to changes in IPES, there is a discrepancy as there is no statistically significant improvement in scores despite a reduction in seizure frequency, as higher impact scores have been demonstrated to be associated with higher seizure frequency. 30 A reason for this could be the broad range in seizure frequencies assessed within the context of this analysis whereby reductions in seizure frequency in those with a high seizure burden at baseline are masked within the context of the IPES by those who had limited impact from their epilepsy at baseline. However, the questionnaire has not been validated to assess whether changes in seizure frequency reported by the same individual are associated with changes in IPES scores. It is important to continue to assess treatment outcomes in children with TRE using an assessment of seizure frequency, in addition to a global assessment of the quality of life, such as IPES to monitor response to treatment holistically.

The doses of CBD utilized in this case series are similar to those utilized in randomized controlled trials for CBD isolates as an adjunctive therapy for TRE, which are between 10 and 20 mg/kg/d. 14 Those being treated with CBD isolates in this case series were maximally titrated to 14.1 mg/kg/d, on average, whilst those on broad-spectrum CBD and combination treatment with Δ9-THC were on lower daily dosing of CBD. There is skepticism about the presence of an entourage effect of the constitutive pharmaceuticals within CBMPs, particularly in the setting of epilepsy. 36 In the present study the reduction in seizure frequency was comparable between those treated with CBD isolate oils and broad-spectrum CBD. This suggests, that despite preclinical evidence suggestive of anticonvulsant effects of minor cannabinoids, terpenes, and flavonoids these had little additional effect at the doses administered in the present study. 37 However, those prescribed CBMPs containing Δ9-THC did have a statistically significant reduction in seizure frequency. A preclinical evaluation of the interactions between CBD and Δ9-THC suggests that there is a complex dose–response relationship with synergistic effects seen at low doses, with negative effects demonstrated by co-administration at higher doses over long periods. 20 This underscores the need for further clinical evaluation of various CBMPs through randomized controlled clinical trials, alongside pre-clinical assessment of the mechanism of action in epilepsy.

The proportion of patients affected by adverse effects in this study (45.7%) is again comparable to those reported by both previous case series from the United States (44%) and Israel (46%). 22 23 These each demonstrate acceptable short-term adverse event profiles across the range of CBMPs that may be utilized in the setting of TRE in children. However, these have not answered the outstanding challenge of determining the long-term safety of Δ ^9^ -THC on the developing brain, which remains the primary concern of many clinicians. Cognitive deficits are already a common comorbidity in children with refractory epilepsy. 38 Despite proposed mechanisms for epilepsy causing cognitive deterioration through damage secondary to excitotoxicity during prolonged seizures, there is no clear evidence to suggest seizures directly cause cognitive decline. 38 Real-world evidence collection through the UK Medical Cannabis and other sources is an important aspect of rigorous pharmacovigilance of CBMPs. 39 Notably, these approaches should be adopted in other anti-seizure medications as well, where the effects on neurodevelopment and cognition with chronic administration are also unclear. 40 41 Whilst this evidence is awaited, clinicians must balance the risk of unknown effects on neurodevelopment with the increased risk of SUDEP, status epilepticus, and psychological comorbidity in patients with persistent seizures refractory to treatment. 4 5

With this case series the inherent limitation is the lack of a comparator arm. Consequently, it is challenging to identify the causative mechanism behind any associated outcomes following the commencement of CBMP therapy. This methodology contrastingly increases the external validity of the study to real-life practice. This is particularly beneficial as a pharmacovigilance strategy to assess the adverse events associated with long-term treatment. This study is affected by a limited sample size. However, the results can be utilized to inform a post-hoc power calculation to inform future studies. Incorporating results from placebo arms of trials in similar populations could similarly inform future randomized controlled trials. The study introduces bias as all patients are treated at Sapphire Medical Clinics, a private medical provider, paying for both clinical consultations and prescriptions. This may produce an expectation of improved outcomes, leading to significant risk of bias. Furthermore, the recording of seizure frequency was not performed utilizing a seizure frequency diary, therefore, the accuracy of the reported changes by families cannot be verified fully. Similarly, all adverse events were considered and there was no attempt to determine which were drug-related which may lead to an over-reporting of adverse events. The discordance in IPES scores and reduction in seizure burden is of concern and is a key reason as to why randomized controlled trials are required to assess CBMPs in this population to assess the veracity of the signals seen in observational data such as this. Finally, a significant limitation is incomplete follow-up data for all patients which may exacerbate selection bias.

## Conclusion

The results show a promising signal towards the effectiveness of CBMPs in children with TRE, particularly in the cohort of patients treated with Δ9-THC. These data should be contextualized within the limitations created by selection bias and the lack of an appropriate control arm for treatment meaning that causation cannot be determined, and it is not possible to negate any placebo effect. Moreover, it was not possible to assess which patients may be most likely to benefit from therapy with CBMPs. The results from this study could be utilized in the design of future phase II randomized controlled trials, particularly for dosing regimens. The short-terms adverse effects appear well-tolerated, but the long-term effects of CBMPs on neurodevelopment are still unknown. The UK Medical Cannabis Registry will form an important component of a pharmacovigilance strategy that will contribute to the long-term data in this patient population.
