# Supplementary material for: Clinical Outcome Data of Children Treated with Cannabis-Based Medicinal Products for Treatment Resistant Epilepsy—Analysis from the UK Medical Cannabis Registry
Source: Neuropediatrics. 2023 Jan 30;54(3):174–81. doi: 10.1055/a-2002-2119 (PMC10166640; doi:10.1055/a-2002-2119)
Supplement: Supplementary file 1 — Supplementary Material [file 10-1055-a-2002-2119-s1020223339oa.pdf]

Supplementary Table S1 Maximum tolerated mean daily dose for each therapy

	CBD Isolate ($n = 19$)	CBD Broad Spectrum ($n = 17$)	CBD & Δ^9-THC ($n = 17$)
Cannabidiol dose (mg/kg/d)	14.1 ± 5.3	5.7 ± 4.1	8.7 ± 4.3
Tetrahydrocannabinol dose (mg/kg/d)	N/A	N/A	0.4 ± 0.3

Abbreviations: CBD, cannabidiol; Δ^9-THC, delta-9-tetrahydrocannabinol.
Note: Data displayed as mean ± standard deviation.

Supplementary Table S2 Number of prescriptions per individual CBMP

Prescription data	Number of prescriptions	CBD dose	Δ9-THC dose
Adven 100 mg CBD/5 mg full spectrum Δ9-THC/mL oil	5	100 mg/mL	5 mg/mL
Adven 150 mg/mL CBD isolate oil	3	150 mg/mL	0 mg/mL
Adven 20 mg/mL Δ9-THC full spectrum oil	2	20 mg/mL	0 mg/mL
Adven 50 mg/mL CBD broad spectrum oil	21	50 mg/mL	0 mg/mL
Bedica <1% CBD/2% Δ9-THC oil	4	<10 mg/mL	20 mg/mL
Bedrolite 10% CBD/< 1% Δ9-THC	6	100 mg/mL	<10 mg/mL
Epidyolex/CBD isolate 100 mg/mL oil	1	100 mg/mL	0 mg/mL
NOIDECS C20, Celixir 20 186.4 mg CBD/9 mg Δ9-THC /mL oil	5	186.4 mg/mL	9 mg/mL

Supplementary Table S3 Comparison of mean difference in Impact of Pediatric Epilepsy Score at each follow-up between those previously naïve to treatment and those who had previously commenced treatment

Follow-up Month	Previously naïve to treatment	Commenced treatment prior to enrollment	*p*-Value
Month 1	−1.9 ± 3.5	−0.9 ± 4.7	0.605
Month 3	−0.9 ± 6.5	−1.2 ± 9.8	0.962
Month 6	−6.7 ± 5.0	−1.3 ± 6.8	0.233

Supplementary Table S4 Comparison of mean difference in Impact of Pediatric Epilepsy Score at each follow-up according to maximally tolerated therapy

Follow-up month	Mean difference CBD isolate	Mean difference CBD whole spectrum	Mean difference CBD and	*p*-Value
Month 1	−3.9 ± 3.8	−4.5 ± 8.0	0.1 ± 4.5	0.280
Month 3	4.2 ± 2.4	−2.8 ± 5.6	−1.7 ± 10.8	0.648
Month 6	−7.4 ± 7.5	−5.1 ± 5.5	−1.7 ± 6.9	0.598

Supplementary Table S5 Adverse events reported by participants prescribed CBD and Δ9-THC CBMPs ($n = 17$)

Adverse events	Mild	Moderate	Severe	Debilitating/Life threatening	Total
Fatigue	5	2	0	0	7 (41.1%)
Seizure frequency increased	0	1	0	0	1 (5.9%)
Sepsis	0	0	0	1	1 (5.9%)
Somnolence	0	1	0	0	1 (5.9%)
Weight loss	0	0	1	0	1 (5.9%)
Total	5 (29.4%)	4 (23.5%)	2 (11.8%)	1 (5.9%)	11 (64.7%)

 © 2023. The Author(s).
